# Transcriptome analysis of *Drosophila melanogaster* laboratory strains of different geographical origin after long‐term laboratory maintenance

**DOI:** 10.1002/ece3.6410

**Published:** 2020-06-08

**Authors:** Mikhail Zarubin, Alena Yakhnenko, Elena Kravchenko

**Affiliations:** ^1^ Molecular Genetics Group Dzhelepov Laboratory of nuclear problems Joint Institute for Nuclear Research Dubna Russia; ^2^ Laboratory of Analytical and Bioorganic Chemistry Limnological Institute Siberian Branch of the Russian Academy of Science Irkutsk Russia

**Keywords:** adaptation, complex traits, differentially expressed genes, *Drosophila*, gene expression, genetic variation, global warming, transcriptome

## Abstract

Positive selection may be the main factor of the between‐population divergence in gene expression. Expression profiles of two *Drosophila melanogaster* laboratory strains of different geographical origin and long‐term laboratory maintenance were analyzed using microchip arrays encompassing probes for 18,500 transcripts. The Russian strain D18 and the North American strain Canton‐S were compared. A set of 223 known or putative genes demonstrated significant changes in expression levels between these strains. Differentially expressed genes (DEG) were enriched in response to DDT (*p* = .0014), proteolysis (*p* = 2.285E−5), transmembrane transport (*p* = 1.03E−4), carbohydrate metabolic process (*p* = .0317), protein homotetramerization (*p* = .0444), and antibacterial humoral response (*p* = 425E−4). The expression in subset of genes from different categories was verified by qRT‐PCR. Analysis of transcript abundance between Canton‐S and D18 strains allowed to select several genes to estimate their participation in latitude adaptation. Expression of selected genes was analyzed in five *D. melanogaster* lines of different geographic origins by qRT‐PCR, and we found two candidate genes that may be associated with latitude adaptation in adult flies—*smp‐30* and *Cda9*. Quite possible that several alleles of these genes may be important for insect survival in the environments of global warming. It is interesting that the number of genes involved in local adaptation demonstrates expression level appropriate to their geographical origin even after decades of laboratory maintenance.

## Graphical abstract

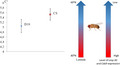

## 1 INTRODUCTION

Different strains of the same species can show a wide range of phenotypic variation concerning all aspects of their life cycle. The study of the basis of this diversity is important for understanding metabolic pathways, longevity, and adaptive evolution and contributes to the improvement of various parameters of human health and life. For example, a few researches using different strains of classic molecular genetics object *Drosophila melanogaster* and recent genome‐wide association studies demonstrated interesting results in genetics of different complex traits (Ayroles et al., 2009), longevity (Moskalev et al., 2019), energy metabolism and respiration (Montooth, Marden, & Clark, 2003), body weight and metabolic rate (Jumbo‐Lucioni et al., 2010)^,^ and energy stores (Klepsatel, Wildridge, & Gáliková, 2019). In addition, examinations of variation in transcript abundance between populations provided information about evolutionary processes such as migration, selection, mutation accumulation (Hsieh, Passador‐Gurgel, Stone, & Gibson, 2007), and parallel evolution (Zhao & Begun, 2017; Zhao, Wit, Svetec, & Begun, 2015).

According to some studies *D. melanogaster* expanded from sub‐Saharan Africa at the recession of the last ice age time from 12,000 till 19,000 years ago (David & Capy, 1988; Li & Stephan, 2006) assuming ten generations per year (Stephan & Li, 2007; Thornton & Andolfatto, 2006) with a subsequent colonization of North America has been occurring in the past 500 years (Stephan & Li, 2007). Most likely that there was a single “out‐of‐Africa” population bottleneck event (Henn, Cavalli‐Sforza, & Feldman, 2012)^,^ and the genome‐wide studies of *D. melanogaster* populations from Africa, North America, Europe, Asia, and the South Pacific confirmed these assumptions (Arguello, Laurent, Clark, & Gaut, 2019). In *D. melanogaster,* latitudinal clines were observed for a large number of traits, for instance, cell membrane plasticity (Cooper, Hammad, & Montooth, 2014), lifespan, lifetime fecundity, heat and cold resistance (Schmidt & Paaby, 2008), and ethanol tolerance (Cohan & Graf, 1985). It is also known that the frequency of certain alleles of genes increases (*Adh‐F*, *Aldh‐Phe*, *Tpi‐F*, *Pgd‐F*) (Berry & Kreitman, 1993; Fry, Donlon, & Saweikis, 2008; Oakeshott et al., 1982; Oakeshott, McKechnie, & Chambers, 1984) and decreases (*EstC^3^*, *Odh‐S*, *LapD^3^*) (Oakeshott, Gibson, Willcocks, & Chambers, 1983; Singh, Hickey, & David, 1982) with latitude. However, a significant part of alleles involved in latitude adaptation is not known.

Several *Drosophilidae* strains of different geographical origin were previously compared at the DNA or RNA levels (USA (Fabian et al., 2012), USA–Australia (Reinhardt, Kolaczkowski, Jones, Begun, & Kern, 2014), USA–Panama (Zhao et al., 2015), and Western European (the Netherlands)–African (Zimbabwe) (Hutter, Saminadin‐Peter, Stephan, & Parsch, 2008) populations), and these studies demonstrated interesting patterns of local adaptation through gene expression differentiation, including sex‐biased gene expression. For instance, local adaptation between African and European populations of *D. melanogaster* mainly manifested increased expression of the gene *Cyp6g1*, responsible for insecticide DDT protection for European populations, as well as genes involved in olfaction and the detection of chemical stimulus (Müller et al., 2011). However, there are data neither at the genomic nor transcriptomic levels obtained from Eastern European (Russia) strains of *D. melanogaster*.

One of the main parameters for adaptation is temperature. In addition, one of the most significant things linked with the problem of global warming (IPCC, 2014) is the extinction of insect species that cannot acclimate to temperature increase (Sánchez‐Bayo & Wyckhuys, 2019). Since insects play critical role in many ecosystems for maintaining biodiversity and stable existence of other species of living organisms, the study of insect adaptations to warmer environments becomes increasingly more important. Understanding of adaptation mechanisms at the genetic and metabolic levels allows predicting the extinction of some insect species during global warming and probably preventing it. Therefore, it seems important to compare Russian (Domodedovo 18) and USA (Canton‐S) *D. melanogaster* strains that lived in different environmental conditions (for instance the average annual temperature in the Canton (Ohio, USA) is about 5,5°C higher than in Domodedovo (Moscow region, Russia)), to obtain new information about transcript abundance between them in case if the long‐term laboratory maintenance keeps the differences between these two lines. D18 (Domodedovo 18) strain of *D. melanogaster* was collected in 1957 by N. P. Dubinin and colleagues from the natural environment in Russia, Moscow region, Domodedovo. The Canton‐Special (Canton‐S) strain was established by C. B. Bridges from wild type flies from Canton region (Ohio, USA) in 1920s, and it is probably one of the most widely used *D. melanogaster* lines. Therefore, the comparison of transcriptome profiles between the two laboratory lines will also allow planning experiments with taking into account the differences in their gene expression levels.

Both lines are highly inbred and under identical conditions any possible phenotypic and transcriptomic differences between them are caused by genetic backgrounds that were previously formed under influence of evolutionary factors and adaptations to dissimilar environment and to some extent during neutral evolution and random genetic drift. Additional moderate contributions can also be made by spontaneous mutations accumulated during maintenance with the point mutation rate 2.8 × 10^−9^ per site per generation and deletion mutation rate of 1.2 × 10^–9^ (Keightley, Ness, Halligan, & Haddrill, 2014).

As shown in recent studies of H. J. Maclean et al. (Maclean, Kristensen, Sørensen, & Overgaard, 2018), laboratory populations of *D. melanogaster* are not fundamentally different from field populations in basic physiological and ecological traits (in particular, in fecundity and developmental time, life span, moving activity level, standard metabolic rate, tolerance to desiccation, starvation, and heat tolerance) and laboratory‐maintained *D. melanogaster* strains can be used in ecological, physiological, and evolutionary comparative experiments. Similar data were obtained by Griffiths, Schiffer, and Hoffmann (2005) suggested that adaptation to laboratory conditions does not interfere with detection of geographical patterns in fact.

Moreover, the use of highly inbred laboratory lines allows to diminish the effects of genetic heterogeneity that observed in wild populations and amplify the impact of studied loci that exhibit different expression levels between strains (Wills, Phelps, & Ferguson, 1975).

Therefore, the whole transcriptome analysis of various *D. melanogaster* laboratory lines can provide new information about local adaptation processes and makes it possible to compare them with the few data obtained from natural populations.

Here, we report the microarray analyses on two laboratory *D. melanogaster* lines of different latitude origin to estimate number and functional affiliation of differentially expressed genes after decades of laboratory maintenance and try to find genes that could be important for adaptation to various environmental conditions and their expression levels can be used as molecular markers of low‐ and high‐latitude *D. melanogaster* populations.

## 2 MATERIALS AND METHODS

### 2.1 *D. melanogaster* stocks and maintenance

We used wild type strains of *D. melanogaster*: Canton‐S (obtained from Prof. Gvozdev V.A., Institute of Molecular Genetics of Russian Academy of Sciences), Oregon‐R (obtained from Dr. Kravchuk O.I., Koltzov Institute of Developmental Biology of Russian Academy of Sciences), D18, Ultuna, and GoS (obtained from Dr. Alexandrov I.D JINR). The strains were cultured on a standard cornmeal‐molasses medium with antibiotic and maintained at 25°C (at synchronized 12:12 hr Light:Dark, 60% humidity).

### 2.2 RNA extraction

Total RNA was extracted from fifteen 2–3 days adult flies of both sexes using TRIzol Reagent according to the manufacturer's protocol. The integrity of RNA was verified using a QIAxcel Advanced System (Qiagen). RNA extraction from Canton‐S and D18 flies was performed at the same time with a one week difference between repeats.

### 2.3 Microarray analysis

cRNA was prepared with GeneChip® 3’ IVT PLUS Reagent Kit (Applied Biosystems, Thermo Fisher Scientific Inc.) according to the manufacturer's protocol. The GeneChip Drosophila Genome 2.0 Arrays (Applied Biosystems, Thermo Fisher Scientific Inc.) were hybridized, stained, and washed according to the manufacturer's protocol and scanned with Affymetrix 3,000 7G scanner. The data set includes the scans of six GeneChip™ Drosophila Genome 2.0 Arrays (designed using Dmel Release 3.1), representing of Canton‐S or D18 lanes in three independent biological repeats. All data were submitted to NCBI GEO database, under accession number GSE146725.

CEL files obtained after scanning were analyzed using the Transcriptome Analysis Console software version 4.0.2 (reference Drosophila melanogaster genome release 3.1). Background correction and normalization were made by RMA algorithm using default parameters. A probe was selected to be differentially expressed if its *p*‐value was < .01 and its mean fold change value across three replicates was more than twofold. Gene ontology analysis for enrichment of biological processes was done by DAVID version 6.8 (data base of all annotated genes was used as a background list, enrichment cut off *p* < .1), up‐ and downregulated genes were analyzed separately. In addition, for searching GO terms for individual genes FlyBase was used.

### 2.4 DNA extraction and sequencing

Total DNA was isolated with standard phenol–chloroform‐based extraction, and PCR fragment of mtDNA was obtained with primers to cytochrome c oxidase subunit I COIF 5′‐CCAGCTGGAGGAGGAGATCC‐3′ and COIR1 5′‐GAGTTCCATGTAAAGTAGC‐3′ (Ilinsky, 2013). Sanger sequencing was performed using the BigDye Terminator v3.1 Cycle Sequencing kit (Applied Biosystems, Thermo Fisher Scientific Inc.) and SeqStudio Genetic Analyzer (Applied Biosystems, Thermo Fisher Scientific Inc.).

### 2.5 Quantitative RT‐PCR

mRNA was treated with dsDNase and converted into cDNA using QuantiTect Rev. Transcription Kit (Qiagen GmbH) and then used for qPCR with iTaq Universal SYBR Green Supermix (Bio‐Rad) on a CFX96 Touch Real‐Time PCR Detection System (Bio‐Rad). The data from three independent biological replicates were analyzed with the Bio‐Rad CFX manager 3.1 software, fold changes in mRNA expression profiles of target genes were evaluated using the 2^‐ΔΔCt^ method. Ras64B, GAPDH, and RpL32 were used as reference transcripts for normalization. The reference and target primers used for the analysis are listed in Table S1.

### 2.6 Climbing assay

In all assays, the day before the climbing tests groups of 5 males and 5 females (2 days old) was placed to new food vials. On the day of experiment, each group was sequential transferred in an empty 50 ml glass graduated cylinder marked at the 8 cm from the bottom with a black line. Flies tapped down to the cylinder bottom had 10 s to climb. The number of flies that passed the 8 cm black line in 10 s was recorded as a percentage of flies above the target line. Three biological replicates with 20 groups (200 flies) in each were run for D18 and Canton‐S strains in the same ambient light, temperature, and humidity.

### 2.7 Critical thermal minimum

Critical thermal minimum measurements were carried out as described in Andersen et al. (Andersen et al., 2015). Eight biological replicates with ten 3 days old flies (5 males and 5 females) were undertaken for D18 and Canton‐S lines.

## 3 RESULTS

### 3.1 Canton‐S and D18 have different mitotypes

Due to the large variability in *D. melanogaster* mitochondrial DNA (it may reach over 100 SNPs (Zhu, Ingelmo, & Rand, 2014)), Ilinsky (Ilinsky, 2013) offered to analyze two conservative substitutions in COI gene—C/T in positions 2,160 and 2,187 (NC_024511.2). The sequencing of 340 bp fragment of mitochondrial COI gene containing these nucleotides demonstrated the difference between Canton‐S and D18 strains: Canton‐S has mitotype CT and D18–TC.

### 3.2 *Differential gene expression in Canton‐S* versus*. D18*

223 (1.18%) genes were significantly differentially expressed (more than 2‐fold) between Canton‐S and D18 *D. melanogaster* strains. 188 genes (84.3%) were upregulated and 35 genes (15.7%) were downregulated in Canton‐S versus. D18.

The differentially expressed genes are associated with a number of functions and pathways including oxidation–reduction process, mitochondrial electron transport, metal ion transport, ion homeostasis, locomotion, cellular response to reactive oxygen species, negative regulation of gene expression, proteolysis, lipid metabolic process, cold acclimation, transmission of nerve impulse, chitin biosynthesis, and others (Table S2). We used DAVID analysis (version 6.8) for all differentially expressed genes to identify significantly enriched functional categories (Table 1).

**TABLE 1:** Biological processes overrepresented among Canton‐S upregulated (red) and downregulated (blue) genes

Gene Ontology (GO) biological process	*p*‐Value
GO:0,006,508 ~ proteolysis (*Ance, CG11841, CG11912, CG16749, CG17475, CG18417, CG30090, CG32369, CG3739, CG3775, CG42370, CG4653,CG6041, CG6337, Jon74E, Phae1, Phae2, Ser6*)	2.2854146441050436E−5
GO:0,055,085 ~ transmembrane transport (*CG14857, CG15553, CG17751, CG32053, CG32054, CG42235, CG5002, CG6125, Mdr50, Zip102B, Zip71B, mfrn*)	1.0317887452110587E−4
GO:0,005,975 ~ carbohydrate metabolic process (*CG16965, CG31414, Cht8, Cda9, GNBP3*)	.03174294986246115
GO:0,051,289 ~ protein homotetramerization (*CG16985, CG16986*)	.04435597930146445
GO:0,019,731 ~ antibacterial humoral response (*CG33470, IMPPP, PGRP‐LC*)	9.424847441977699E−4
GO:0,046,680 ~ response to DDT (*Cyp12d1‐p, Cyp12d1‐d, Cyp6a14*)	.0013996376177856254
GO:0,045,087 ~ innate immune response (*CG33470, IMPPP, PGRP‐LC*)	.01809381424769528
GO:0,017,085 ~ response to insecticide (*Cyp12d1‐p, Cyp12d1‐d*)	.02766496492413906
GO:0,055,114 ~ oxidation–reduction process (*Cyp12d1‐p, Cyp12d1‐d, Cyp12c1, Cyp6a14*)	.04742516355840346

Besides this among the Canton‐S, differentially expressed genes were upregulated genes from dopamine uptake involved in synaptic transmission (*CG13793, CG33296*), metabolic process (*CG10170, CG32984, Ugt86Dh, CG5724, and CG6429*), sodium‐independent organic anion transport (*Oatp33Eb, Oatp58Da*), cold acclimation (*Hsp26, smp‐30*), oxidation–reduction process (*Cyp6d4, Cyp4ac1, Cyp4p2, CG31674, Cyp28a5, Cyp9b2, Hmgcr, Prx2540‐2, P5cr‐2, and dj‐1β*), transmission of nerve impulse (*Ir76a and axo*), electron transport chain (*RFeSP*), locomotion, and regulation of synaptic growth at neuromuscular junction (*Syn2*). In addition, there are two differentially expressed transcription factors *SmydA‐9* (CG12119) and *sbb* which can potentially regulate the observed alterations in expression between Canton‐S and D18 genes.

Several retrotransposons were significantly differentially expressed too: Retrotransposons *1731* (1625050_s_at) and *springer* (1624543_s_at) were upregulated in Canton‐S strain (fold change 33.93 and 8.73, respectively) whereas retrotransposable element *R2Dm* (1630934_at) and endogenous retroviruses *tirant* (1640955_s_at) demonstrated increased expression level in D18 strain (fold change 1,574 and 1,385, respectively).

### 3.3 Verification of microarray results using quantitative RT‐PCR

**FIGURE 1:**
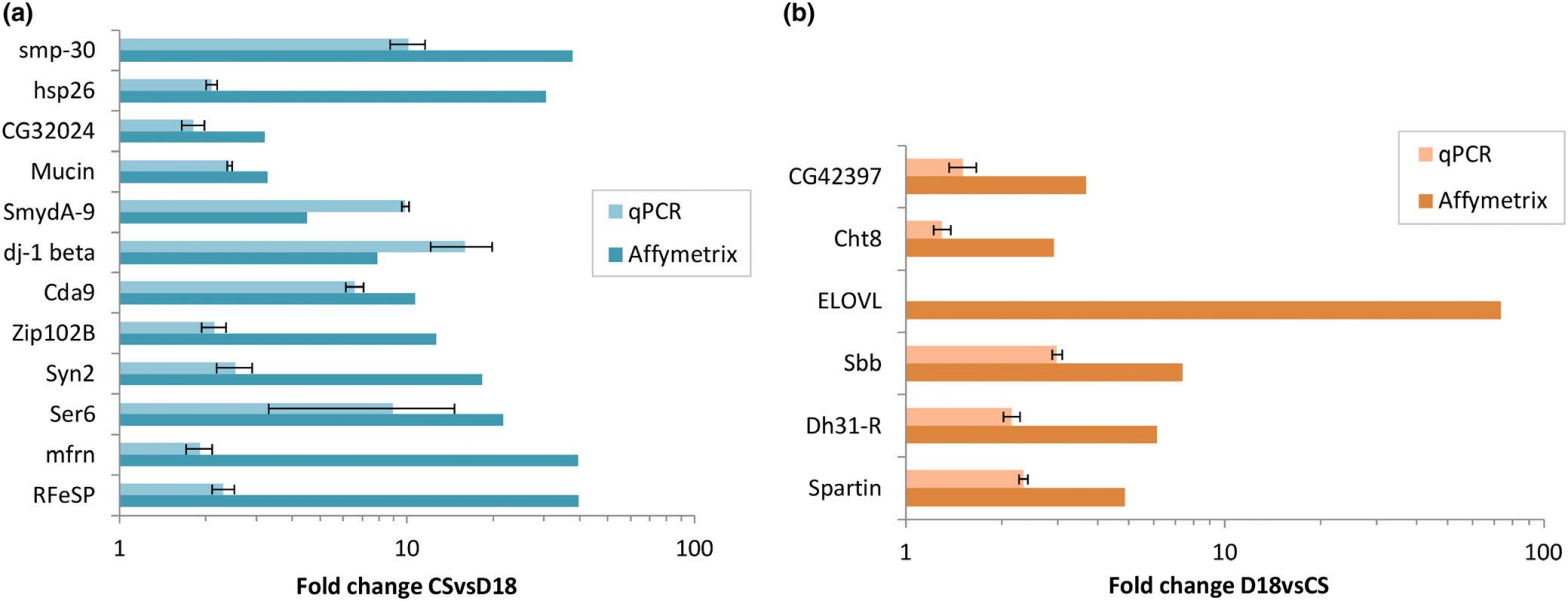
Comparison of the relative expression difference between D18 and Canton‐S *Drosophila melanogaster* laboratory strains obtained by microarray and qRT‐PCR. (a) upregulated genes in Canton‐S strain. (b) upregulated genes in D18 strain. Fold change is shown on a log scale with base 10; *t* test, *p* < .01 for all comparisons except Cht8 (*p* < .05)

To verify the differentially expressed genes between Canton‐S and D18 strains, we randomly selected twelve genes from different biological processes and six genes that may be involved in adaptation (description in Section 3.4) and compared expression levels for these genes using qRT‐PCR. Analysis of differences in gene expression obtained by quantitative RT‐PCR in compare with microarray results is shown in Figure 1.

The results obtained by quantitative RT‐PCR agree with the results obtained by microarrays except *ELOVL* gene—by qRT‐PCR analysis it demonstrated the same level of expression in both lines.

### 3.4 Latitudinal clines of smp‐30 and Cda9 gene expression

We measured level of gene expression using qRT‐PCR for several genes that may be candidates for latitude adaptation for five laboratory *D. melanogaster* lines of different latitude origin: Canton‐S (40°18’N), Oregon (43°13’N), Golden Sands (52°N), D18 (55°26’N), and Ultuna (59°49’N) and compared the fit of regression lines generated between latitude and the expression level for several candidate genes. Six genes were chosen for analyses: *hsp26* involved in response to brief exposure to low temperatures (Qin, Neal, Robertson, Westwood, & Walker, 2005); *smp‐30,* previously it was shown that *smp‐30* demonstrates clinal expression pattern across eastern Australia between 15 and 45°S and involved in cold acclimation (Clowers, Lyman, MacKay, & Morgan, 2010; Lee et al., 2011); *mucin,* mucin protein takes part in several important processes related to adaptation to different environments (protection of epithelium from drying out, pathogens, toxic substances, etc.) (Syed, Härd, Uv, & van Dijk‐Härd, 2008); *ser6*, coding serine protease, it is one of the top differentially expressed genes and may be involved in protein degradation process dependent on geographic variations in insect diet; *Cda9* required for morphogenesis of tracheal network (Luschnig, Bätz, Armbruster, & Krasnow, 2006) which is important for thermoregulation and may be for local adaptation; *syn2* involved in locomotion processes and in regulation of synaptic morphology (Nagai, Hashimoto, & Yamaguchi, 2010) that determine some parameters of adaptive behavior.

**FIGURE 2:**
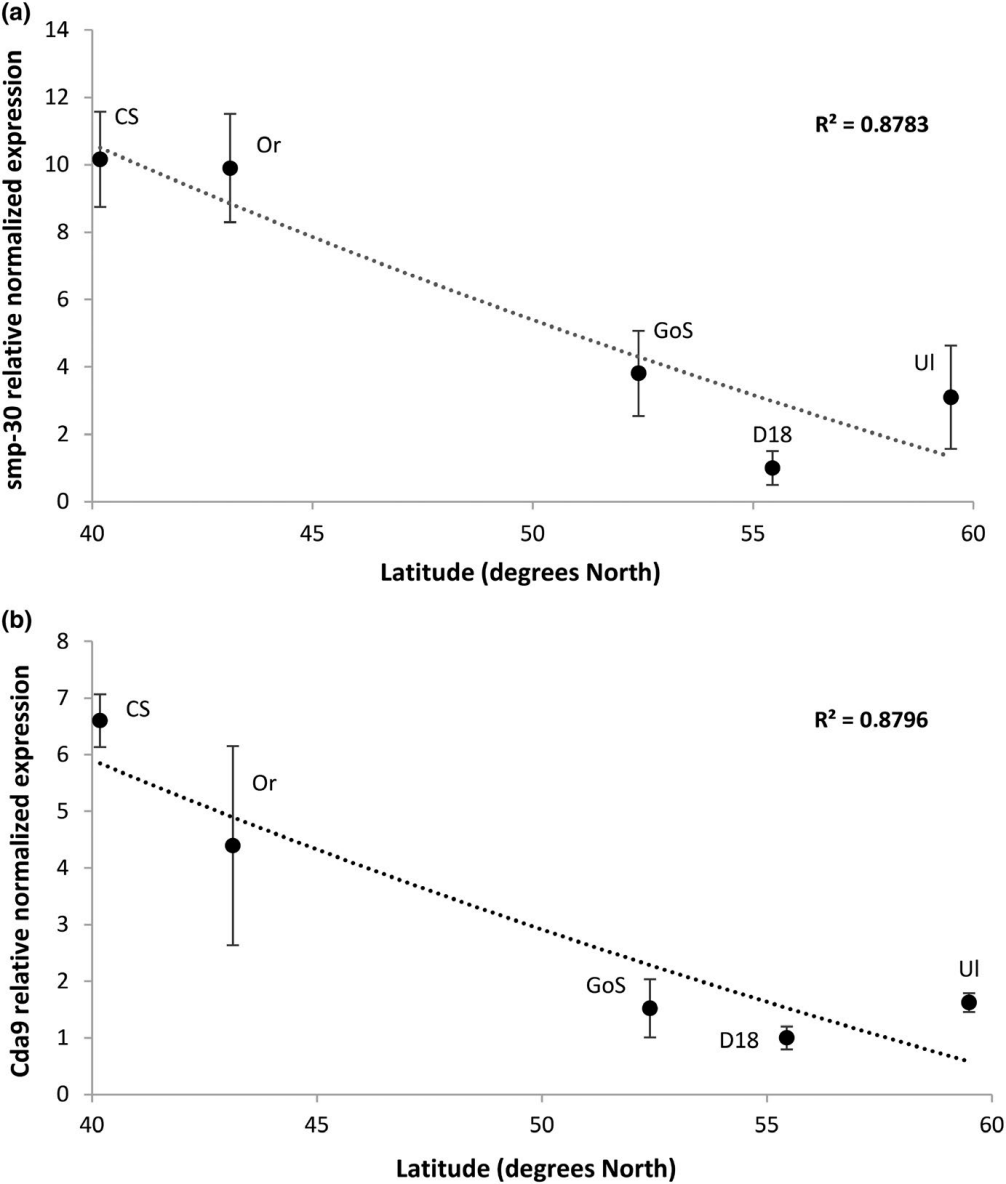
Comparison of the relative expression difference of smp‐30 (a) and Cda9 (b) genes between *Drosophila melanogaster laboratory* strains. CS—Canton‐S, Or—Oregon, GoS—Golden Sands, D18—Domodedovo 18, Ul—Ultuna. D18 expression level was taken as a control sample. The significant linear regression lines are shown

*Ser6, syn2, mucin,* and *hsp26* did not demonstrate latitude dependence of the levels on their expression (data not shown), but a downward trend was observed for *smp‐30* and *Cda9* gene expression (Figure 2).

### 3.5 Climbing ability differs between D18 and Canton‐S strains

**FIGURE 3:**
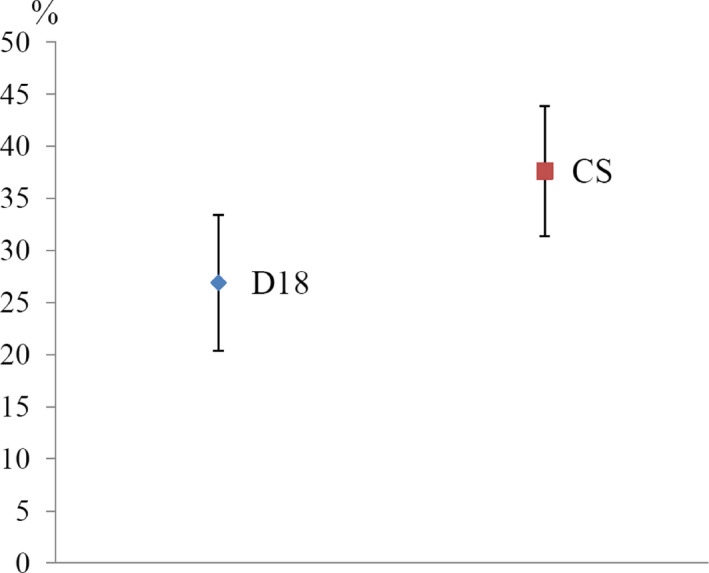
Results of climbing assays. Dat points represent the mean ± standard error of mean (*SEM*) of the percentage of D18 and Canton‐S flies able to climb above 8 cm during 10 s (*t*‐test, *p* < .05)

The results of climbing assays demonstrated strain‐specific climbing ability: 26.9% of D18 flies passed the 8 cm line during 10 s whereas for Canton‐S flies this value was 37.6% (Figure 3). Thus, D18 strain has reduced climbing ability compared with Canton‐S strain.

### 3.6 D18 strain has lower critical thermal minimum (CTmin) than Canton‐S strain

**FIGURE 4:**
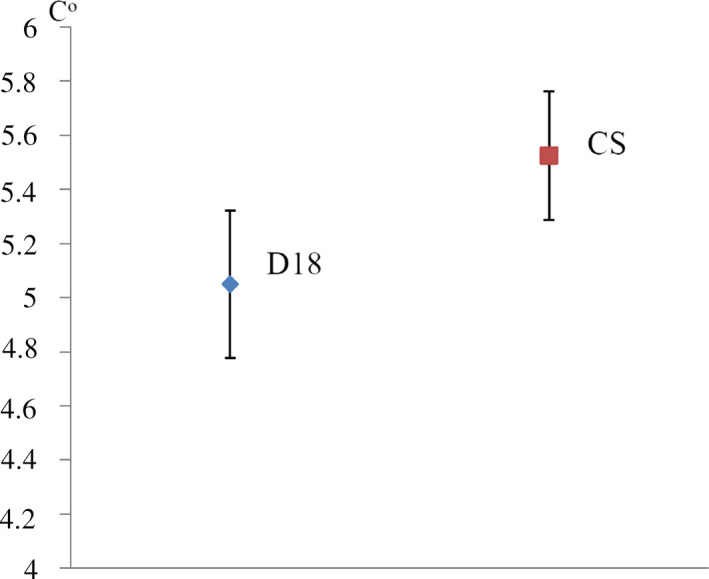
CTmin estimation results. Data points represent the mean of CTmin ± standard error of mean (*SEM*) (*t* test, *p* < .01)

Several phenotypic traits can been used to estimate *D. melanogaster* cold tolerance: critical thermal minimum (CTmin), lower lethal temperature (LTe50), chill coma recovery time (CCRT), and others (Sinclair, Coello Alvarado, & Ferguson, 2015). Here, we measured CTmin (the temperature when flies lose ability to move) that correlated to other parameters of cold tolerance in Drosophila (Andersen et al., 2015). D18 flies demonstrated complete absence of response to stimulation with light and tapping on an average at 5.5°C and Canton‐S flies—at 5.525°C (Figure 4). Thus, we can conclude that by comparison CTmin of D18 and Canton‐S strains CTmin of D18 flies turned out ~0.5°C lower.

## 4 DISCUSSION

In addition to the well‐studied genome, the Canton‐S strain is characterized by a number of phenotypic features (for instance rapid phototaxis (Benzer, 1967) and fast weight gain at young age (Qiu, Xiao, & Meldrum Robertson, 2017)) that makes interesting to compare it with other *D. melanogaster* lines to study the basis of phenotypic diversity between populations with different latitude origin. It is important to note that studies of the adaptive responses to rapid cold stress/heat shock/ desiccation stress and other types of stress reflect only one side of the acclimatization of organisms to various environments, while adaptive processes that occur constantly during long‐term (possibly throughout life) exposure of organisms to different latitude conditions are also necessary for survival and require study. For instance, detailed review was performed by Teets and Denlinger for insect cold acclimation (Teets & Denlinger, 2013) where they substantiated the hypothesis that rapid cold‐hardening and long‐term cold acclimation may be based on different physiological processes and MacMillan et al. gave experimental support for this idea in their study of *D. melanogaster* transcriptome and metabolome during cold acclimation (MacMillan et al., 2016). Here, we investigate the expression variations in the absence of any stress conditions between two wild type strains of *D. melanogaster* of different geographical origin—Canton‐S (low latitude USA population 40° 18′18″ N) and D18 (high latitude Russian population 55°26′24″ N)—and discuss the candidate genes for long‐term latitude adaptation that belong to constantly expressed group.

Sequencing of mtDNA fragment revealed that Canton‐S and D18 strains have different mitotypes, it confirms that they have different origins, and sometimes mitotype can influence some phenotypic traits including energy metabolism and longevity (Towarnicki & Ballard, 2018).

In addition, we observed some phenotypic diversity between D18 and Canton‐S strains: namely differences in pigmentation of female abdominal cuticular structures, critical thermal minimum (CTmin), and climbing ability.

Cold temperature is the environmental parameter that greatly determines the insect ability to adapt to certain geographic conditions. Temperature gradients existing during the day and during the year imply the presence of special mechanisms to maintain homeostasis in small ectotherms and to provide a suitable performance of physiological and metabolic processes. As shown before pigmentation of abdominal segments in *D. melanogaster,* females depend on temperature and are involved in thermoregulation (Gibert, Moreteau, & David, 2000). Canton‐S females do not have pronounced pigmentation in abdominal tergite A7 whereas D18 females have a dark‐colored A7 in agreement with Bogert's rule. Also, D18 strain demonstrated CTmin approximately 0.5°C lower that Canton‐S strain that apparently reflects adaptation to temperature conditions considering that the average annual temperature in Domodedovo (Moscow region) is 4.9°C and in Canton (Ohio) is 9.7°C (data from en.climate‐data.org).

It is also known that locomotor behavior is very important for life activity and adaptation processes but the genetic architecture of this complex trait is largely uncharacterized. Thus, analyses of individual components of motor activity such as climbing ability between different *D. melanogaster* strains followed by transcriptome analysis make possible to describe some part of genetic basis of this complex behavior. Climbing assay is widely used to examinate motor function in *D. melanogaster* (Bartholomew, Burdett, Vandenbrooks, Quinlan, & Call, 2015) including studies of Parkinson's disease (Feany & Bender, 2000). Our results of climbing assay for D18 and Canton‐S strains showed difference in their climbing ability—the number of D18 flies able to climb 8 cm in 10 s was approximately 10% less than this number for Canton‐S flies (*t*‐test, *p* < .05). The observed difference can be due for many reasons, including variations in energy metabolism, functioning of muscle, central nervous system, and peripheral neuron between D18 and Canton‐S strains; however, the transcriptome analysis described below suggests candidate genes that may cause this effect.

Analysis of microarrays data indicated that the expression levels for the most genes between Canton‐S and D18 strains demonstrated similar values. At *p* **‐**value < 0.01 and mean fold change value across three replicates, more than twofold 1.18% of genes were significantly differentially expressed. It is lower than between low‐ and high‐latitude populations of *D. melanogaster* from wild environments where this parameter reached 5.4% at 21°C (Zhao et al., 2015) and approximately 3% at 22°C (Hutter et al., 2008). Highly likely, reduced number of differently expressed genes is connected with decreased genetic heterogeneity in laboratory wild type lines. However, the genes with different level of expression between Canton‐S and D18 strains belong to important metabolic pathways, for instance energy metabolism and carbohydrate metabolic process.

The differentially expressed genes are associated with a number of functions and pathways including oxidation–reduction process, mitochondrial electron transport, metal ion transport, ion homeostasis, locomotion, cellular response to reactive oxygen species, negative regulation of gene expression, proteolysis, lipid metabolic process, and chitin biosynthesis. Our data are highly comparable with functional analysis of genes expressed differentially between African and European populations of *D. melanogaster* where GO overrepresented categories were fatty acid metabolic process, oxidoreductase activity, carbohydrate metabolic process, actin cytoskeleton, and actin filament (Hutter et al., 2008). Several functional groups containing differentially expressed genes may be good candidates for local adaptation, for instance chitin biosynthesis, oxidation–reduction processes, transmembrane transport, etc. It should be noted that the second in the list of GO biological processes overrepresented among Canton‐S upregulated genes is transmembrane transport that may reflect the adjustment of water and salt balance (osmoregulation) in different geographical environments and maintenance of ion homeostasis for life activity according to needs of analyzed *D. melanogaster* strains.

**FIGURE 5:**
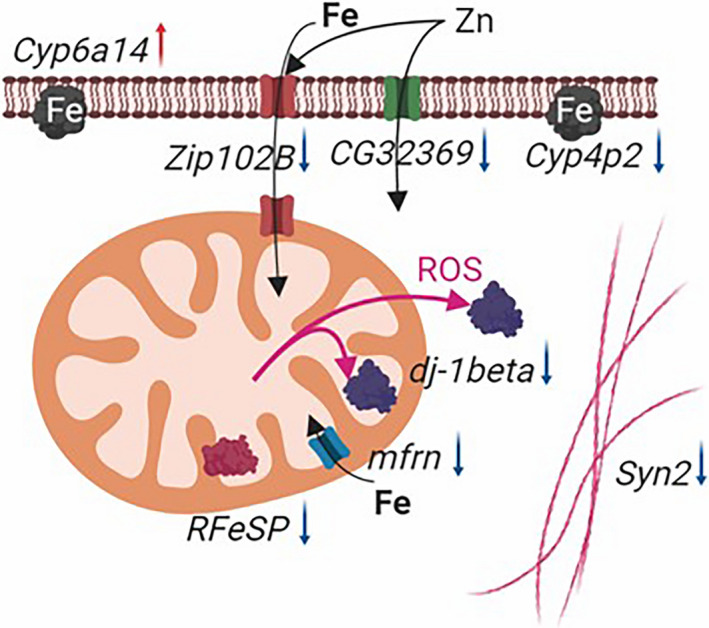
Putative pattern of molecular interconnections between products of up‐ and downregulated (red and blue arrows) D18 genes. ROS—reactive oxygen species

Some of downregulated in D18 genes code proteins with iron transporter activity (*mfrn*, *Zip102B* and probably *CG32369*) or proteins containing iron in active center for oxidoreductase activity (*RFeSP* and *Cyp4p2*). Iron plays a vital role in many metabolic processes and the synthesis of heme, and Fe‐S clusters are coordinately regulated by iron availability (Muckenthaler, Galy, & Hentze, 2008). These results provide possible molecular interconnections between products of five downregulated D18 genes (Figure 5), where possible decreased amount of cytochrome b‐c1 complex subunit Rieske Iron–Sulfur Protein (RFeSP) (mitochondrial location) causes the consistent decreasing of the iron–zinc transporters number. Besides decrease in the number of RFeSP, which is a part of the mitochondrial respiratory chain complex III that is a point of electron leak during electron transport (Jastroch, Divakaruni, Mookerjee, Treberg, & Brand, 2010), causes an decrease of ROS (reactive oxygen species) that in turn reduce dj‐1beta gene expression, that involved in the protection against ROS (Lavara‐Culebras & Paricio, 2007). A decrease of RFeSP amount probably may reflect a general decrease in level of mitochondrial respiratory chain activity. Quite possible that some of these traits may be the result of cooperation work between mitochondrial and nuclear genes (Mossman, Ge, Navarro, & Rand, 2019).

Interestingly that D18 strain had less active *Syn2* gene that takes part in a normal formation of actin‐rich structure, required for eye development (Nagai, Hashimoto, Tanaka, et al., 2010), locomotion, and regulation of synaptic morphology (Nagai, Hashimoto, & Yamaguchi, 2010). It is possible that it is the result of a reduced level of mitochondrial respiratory chain work the effectiveness of which is not enough to maintain the *Syn2*‐dependent processes in D18 at the Canton‐S level (Figure 5). It can be assumed that the lower result of D18 flies compared with Canton‐S flies in climbing test may be due to decreased expression level of *syn2* too. Obtained data suggested that different wild type laboratory strains can significantly differ one from another in important traits, and this fact needs to be considered in comparative experiments.

Other differentially expressed genes encoding iron‐containing proteins belong to cytochrome P450 superfamily: *Cyp4p2, Cyp6d4, Cyp28a5, Cyp4ac1, and Cyp9b2* upregulated in Canton‐S and *Cyp6a14, Cyp12d1‐d, Cyp12d1‐p, and Cyp12c1* upregulated in D18. Perhaps some of these proteins may carry out the same functions in studied strains. It should be pointed out that variations in expression of genes from P450 superfamily also were often observed between field *D. melanogaster* populations (Zhao et al., 2015) and lines (Green, Battlay, Fournier‐Level, Good, & Robin, 2019).

There is, it should be noted, an important difference between expression levels of genes involved in response to DDT and other insecticides. Insecticide resistance can be justified by several factors. Most of the studied populations showed resistance to DDT justified by increased oxidative metabolism associated with the activity of cytochrome p450 monooxygenase. This, in turn, is associated with increased activity of genes of the P450 family, in particular, *Cyp6g1, Cyp6a2,* and *Cyp12d1* located on the right arm of chromosome 2. (Feyereisen, 1999, 2005). Canton‐S had been established before insecticides began to be widely used in agriculture and demonstrated decreased expression levels for *Cyp12d1‐p, Cyp12d1‐d, and Cyp6a14* genes, which can be considered as baseline levels of expression of these genes in the absence of selection pressure associated with the insecticides usage. D18 isolated in the 60s of the 20th century when about 20 thousand tons of DDT were used per year in USSR (Fedorov, 1999). Apparently, an increased expression of *Cyp12d1‐p, Cyp12d1‐d, and Cyp6a14* genes reflects the adaptive processes generated by D18 strain in response to the insecticides usage.

Cold acclimation is an important part of latitude adaptation, according to previously obtained data, and cold acclimation should affect a number of important biological processes including regulation of metabolic balance (Williams et al., 2014), refolding, or stabilizing proteins (Colinet & Hoffmann, 2012) and maintenance of ion and water homeostasis (Macmillan, Andersen, Loeschcke, & Overgaard, 2015). MacMillan et al. revealed that cold acclimation is associated with changes in Na^+^, water, and K^+^ balance (Macmillan et al., 2015) and we found several differentially expressed genes involved in such processes (Nha2—upregulated in D18 flies, CG42235, CG13793, and CG33296—upregulated in Canton‐S flies). Moreover, we observed a number of genes representing biological processes previously identified as candidates for latitude adaptation, for example, coding odorant‐binding protein (Obp56a—upregulated in Canton‐S flies) (Cicconardi et al., 2017), cuticle formation proteins (Cpr65Au—upregulated in Canton‐S flies, Cpr23B—upregulated in D18 flies) (Juneja, Quinn, & Jiggins, 2016), and genes involved in lipid, fatty acid, and steroid metabolism (CG15829, CG31810, and CG31809 upregulated in Canton‐S flies, CG42668 and CG2781 upregulated in D18 flies) (Williams et al., 2014).

Thus, it is obvious that different wild type laboratory lines differ in many important traits among which there is a number mirror adaptive process. Therefore, we tried to isolate genes whose expression depends on latitude and microarray analyses of Canton‐S and D18 strains originated from different latitude provided data for highlighting candidate genes that can be studied individually. Among six selected genes that can be involved in latitude adaptation, two genes (*Cda9* and *smp‐30*) in five studied wild type laboratory *D. melanogaster* lines with different geographical origin demonstrated latitude dependence of the levels on their expression.

Animal body size demonstrated adaptive latitude shift for many species—it smaller in low latitude environment (Bergmann's rule)—and *smp‐30* plays critical role in establishment of wing and body size through negative influences on cell proliferation and tissue size in *D. melanogaster* (Lee et al., 2011). According to the ModECODE data in adults, *smp‐30* exhibited high level of expression in digestive system, carcass, head, and male reproductive tissues and apparently it takes part in calcium homeostasis regulation and negatively affects on cell proliferation (Lee et al., 2011; Yamaguchi, Morooka, Misawa, Tsurusaki, & Nakajima, 2002). Lee et al. found a significant negative latitude cline of *smp‐30* expression in third instar larvae from wild type *D. melanogaster* strains collected across eastern Australia between 15 and 45°S. Consistently with this expectation, we conforming to the laws of nature detected a negative latitude‐dependent change in expression of *smp‐30* between five lines studied in our work and give a new evidence for the clinal pattern of *smp‐30* expression in latitudes higher than 40°N. As we showed, the clinal pattern of *smp‐30* expression persists in adults flies too and the data obtained for *smp‐30* on laboratory lines repeat the trend obtained on the wild populations.

The second gene demonstrated latitude cline expression during our study is *Cda9* coding chitin deacetylase that catalyzes N**‐**deacetylation of chitin to chitosan. Chitin deacetylases (CDAs) are very important for insect growth and development—the decreasing of CDAs level resulted in defective molting at all developmental stages and cuticular abnormalities in multiple insects (Wu et al., 2019). Deacetylated glucosamine residues of chitosane linked with different cuticle proteins to form specific chitin–protein matrices (Tsigos, Martinou, Kafetzopoulos, & Bouriotis, 2000). In *D. melanogaster,* this chitinous matrix formed with the participation of CDAs required for morphogenesis of highly branched tracheal network (Luschnig et al., 2006). Because of tracheal system functions as a respiratory organ in *D. melanogaster,* the increased *Cda9* expression in low‐latitude Canton‐S and Oregon strains may be related to the need to have more extensive tracheal system for thermoregulation and oxygen level control or may be for increased chitin flexibility.

Thus, it can be noted that in five laboratory lines during prolonged living in the laboratory conditions the expression levels of smp‐30 and Cda9 with high probability were not a result of random events (probability of occurrence of such an event 0.03), which implies the possibility of using expression profiles of laboratory lines to search for the genes involved in adaptation processes many years ago. The correlation between differential gene expression and latitude origin of studied *D. melanogaster* laboratory strains may indicate a strong fixation of adaptation patterns in the genome even in the absence of selection pressure.

## 5 CONCLUSIONS

Differences in quantitative and physiological traits between animal populations often reflect adaptive processes. *D. melanogaster* adapted to habitat in widest range of climatic zones makes it very interesting to study local adaptation especially in light of global warming. Numerous studies demonstrated clinal patterns of several traits related to temperature—in particular, body size (Huey, Gilchrist, Carlson, Berrigan, & Serra, 2000) and heat and cold resistance (Hoffmann, Anderson, & Hallas, 2002). Some of studies were carried on using *D. melanogaster* field populations and some on laboratory wild type strains. The questions about effects of long‐term laboratory maintenance on insects and the validity of using laboratory strains in ecology, physiology, and evolution experiments discussed in detail in the work Maclean et al. (Maclean et al., 2018) including analysis of all previous data concerning this topic from various researchers. In sum, in many important traits laboratory lines are representative of their wild founders.

In this work, we moved to the molecular level and showed that laboratory wild type *D. melanogaster* strains with different geographical origin demonstrated difference in the patterns of gene expression even after decades of living in the same laboratory environment. This difference may affect crucial biological pathways, in particular, mitochondrial electron transport, locomotion and resistance to insecticide, which is extremely important to consider when planning experiments with *D. melanogaster* lines. Some of these differentially expressed genes reflect the positive selection between *D. melanogaster* strains and show what traits are important for local adaptation. This is consistent with the data obtained by Maclean et al. evidenced that laboratory‐maintained and freshly collected *D. melanogaster* populations demonstrated generally similar correlations in main physiological and ecological traits (including stress‐tolerance, body size traits, and life history) and lack of change in fundamental characteristics during laboratory maintenance (Maclean et al., 2018). Among six genes with differences in expression level, we identified two genes that may be candidates for latitude adaptation in adult flies—*smp‐30* and *Cda9*. Highly likely, these genes should be taken into consideration when studying insect capabilities adapt to global warming. Obviously that observed alterations in gene expression between Canton‐S and D18 strains are based on different alleles of studied genes or different patterns of gene regulation, which requires further study.

## CONFLICTS OF INTEREST

The authors declare no conflict of interest.

## AUTHOR CONTRIBUTION

**Mikhail Zarubin:** Data curation (equal); Formal analysis (equal); Methodology (equal); Visualization (equal); Writing‐review & editing (equal). **Alena Yakhnenko:** Data curation (supporting); Visualization (equal); Writing‐review & editing (equal). **Elena Kravchenko:** Conceptualization (lead); Data curation (equal); Formal analysis (equal); Methodology (equal); Supervision (lead); Validation (lead); Visualization (supporting); Writing‐original draft (lead); Writing‐review & editing (supporting).

## Supporting information

Table S1

## Data Availability

Microarray data: NCBI GEO database, accession number GSE146725. Supplementary data: DRYAD https://doi.org/10.5061/dryad.zs7h44j5h
