# Supplementary material for: Transcriptome analysis of Drosophila melanogaster laboratory strains of different geographical origin after long‐term laboratory maintenance
Source: Ecol Evol. 2020 Jun 8;10(14):7082–93. doi: 10.1002/ece3.6410 (PMC7391317; doi:10.1002/ece3.6410)
Supplement: Supplementary file 1 — Table S1 [file ECE3-10-7082-s001.pdf]

Table S1. List of primers used for qRT-PCR

Primer	Sequence
Zip102b-rt-Fw	GATGGAGTTGCATTGGGAGC
Zip102b-rt-rev	TAACGTGTCCTTCTGCTCCT
Cda9-rt-Fw	TAGCGCTACATTCGGTGACC
Cda9-rt-rev	CGAAGTTTGGAGGAACGGCA
Dj-1beta-rt-Fw	CTCGCGGGATGTGCAGAT
Dj-1beta-rt-rev	CACAGATGGCGGCGATGA
SmydA-9-rt-Fw	GCAGATGATGAAGTCGCAGG
SmydA-9-rt-rev	TGGGTAATGTCACGGCTGTA
Spartin-rt-Fw	ACACCTTCGGCATTCCAGTA
Spartin-rt-rev	TCTCTGCTCGACTTTCCTCC
Dh31-R-rt-Fw	ACACTACTTCCTCCTGTCCA
Dh31-R-rt-rev	GCCATTTGACCAGCCTCTTC
Sbb-rt-Fw	TATCTGCTGATGTTGCTGTTG
Sbb-rt-rev	AGTATTGCTGTGCCGAGAAG
ELOVL-rt-Fw	GTTCGTCCTGGTTATGGTGC
ELOVL-rt-rev	CATCGCGTTTCACATAGGCA
GAPDH-rt-Fw	CAAGTTGTGGCGTGATGGAC
GAPDH-rt-rev	ATCATAGGACGCACCCTTGC
RFeSP-Fw	ATGATGAACGCCGTGTCGCGTG
RFeSP-rev	TGACGGCCACGCCGCTCGCCTTC
mfrn-rt-Fw	CCGAGGACTACGGATGAGGA
mfrn-rt-rev	CGCTCGTAGGGACTCTTCAC
Mucin-rt-Fw	ATGATTGCACAAAGCACAGGTATC
Mucin-rt-rev	ATTCTTCATCTCTACCGCCTTAC
ser6-rt-Fw	ATTCTGTCCGCGAGCATTCA
ser6-rt-rev	ACACTGCTGCCTGGTTATCG
syn2-rt-Fw	ACCCGACTCGGACAACGAT
syn2-rt-rev	GGCAACCACGTCGGTCCA
smp-30-rt-Fw	ATCGACACCAACAACCACGA
smp-30-rt-rev	ATTGCCATCGGTGTCTACGG
Cht8-rt-Fw	ATGTGGAGCGTATCTGGTT
Cht8-rt-rev	ATGCTGCCATCAGGATTAC
42397-rt-Fw	CCTCCAGTCCTGTGTGCTGATG
42397-rt-rev	GATATGTCCAGACGGCCTCTA
32024-rt-Fw	CTGAGAGCAATGTAGCCTGCG
32024-rt-rev	CATGTATACACGGTACCTGTAG
Hsp26-rt-Fw	ATGTCGCTATCTACTCTGCT

Hsp26-rt-rev	CCAGTCCAAGCTCGTAGATG
rpl32-rt-Fw	GTTCGATCCGTAACCGATGTTG
rpl32-rt-rev	CCAGTCGGATCGATATGCTAAG
ras64B	(Erokhin et al., 2013)

Erokhin, M., Davydova, A., Parshikov, A., Studitsky, V.M., Georgiev, P., Chetverina, D. Transcription through enhancers suppresses their activity in *Drosophila*. 2013. Epigenetics Chromatin. 6, 31. doi: 10.1186/1756-8935-6-31.
